# Advances in Patient Monitoring Systems for Prehospital and Resource-Limited Settings

**DOI:** 10.5811/westjem.47239

**Published:** 2026-02-10

**Authors:** Justin E. Markel, Tanner Smida, Brad Price, James Bardes

**Affiliations:** * Huntington Hospital, Department of General Surgery, Pasadena, California; † West Virginia University School of Medicine, Department of Trauma and Acute Care Surgery, Morgantown, West Virginia; ‡ West Virginia University, Department of Business and Economics, Morgantown, West Virginia

## Abstract

**Introduction:**

Vital sign monitoring is essential to the management of critically ill and injured patients. Recent advances in patient monitoring systems have the potential to improve outcomes by providing real-time data and predictive insights, which are particularly valuable in prehospital and resource-limited settings. We conducted a systematic review of the literature to assess the capabilities, performance, and clinical impact of patient monitoring technologies designed for these environments.

**Methods:**

In accordance with Preferred Reporting Items for Systematic Reviews and Meta-Analyses guidelines, we conducted a systematic review using PubMed and Scopus search engines on studies published between 2018–2022 that proposed or tested novel patient monitorint systems with utility in prehospital or resource-limited settings. Two reviewers independently screened studies, and discrepancies were resolved by a senior author. Of 217 studies identified in the search, 40 met the proposed inclusion criteria.

**Results:**

Compared to standard platforms, wearable and contactless systems for patient monitoring demonstrated high accuracy but with delayed responsiveness and less reliable temperature measurements. Artificial intelligence (AI)-based platforms consistently outperformed well-accepted scoring systems in predicting outcomes such as mortality, intensive care unit (ICU) admission, and clinical decompensation. In this review we summarize proposals for prototypes of integrated patient monitoring systems that combine biosensors, AI algorithms, global positioning system, and wireless communication designed to facilitate triage in prehospital settings, and we then compare their components. Various platforms were piloted and demonstrated minimal disruption to workflow and positive user feedback, although most lacked comprehensive cost analyses.

**Conclusions:**

Emerging patient monitoring system technologies may enhance remote triage and care delivery, particularly in resource-limited settings. However, significant barriers remain, including cost, limited testing in real-world environments, and the lack of higher tiers of evidence. Future efforts should prioritize field-based testing, usability in low-resource settings, and cost-effectiveness analyses to guide clinical adoption.

## INTRODUCTION

Effective triage in prehospital settings hinges on accurate physiologic assessment.^1^–^3^ Vital signs (heart rate [HR], blood pressure [BP], pulse oximetry [SpO_2_], body temperature, respiratory rate [RR], and end-tidal carbon dioxide (ETCO_2_]) are crucial in managing critically ill patients. While they are routinely used to guide clinical decision-making in the hospital, emerging evidence suggests additional utility in the prehospital setting. For patients in hemorrhagic shock, studies have shown that targeting lower systolic BP and mean arterial pressure leads to improved survival, and routing patients to trauma centers based on specific vital sign parameters improves outcomes.^4^–^7^ Similarly, an association between worse outcomes and prehospital hypotension, hypoxia, and hyperventilation has been found in patients with traumatic brain injuries.^8^–^13^ In 2019 Spaite et al published some of the first evidence that regimented management of SBP, SpO_2_, and ETCO_2_ in the prehospital setting could improve outcomes in patients with severe traumatic brain injuries.^14^,^15^ Similar findings have since emerged regarding systolic BP^16^–^20^ and SpO_2_^18^,^21^,^22^ in out-of-hospital cardiac arrest patients. Beyond traditional vital signs, other novel objective physiologic metrics have shown promise in predicting post-arrest and post-traumatic injury outcomes, including the need for life-saving interventions.^23^–^28^ As time is a critical factor in efficient triage and transfer, these findings underscore the utility of using objective patient measurements from first contact with patients.

Due to constraints in equipment, personnel, and time, the prehospital environment is often resource-limited; however, it is not the only context in which patient monitoring poses challenges. Rural and under-resourced health systems also face infrastructural and logistical barriers that limit the application of traditional hospital-based monitoring technologies. Therefore, this review includes studies proposing or evaluating patient monitoring systems in both prehospital and resource-limited settings, including rural, remote, or underserved regions. Our aim in this review was to synthesize innovations published between 2018–2022 that may be adaptable across these overlapping domains, where rapid triage and early physiologic assessment can improve outcomes despite constrained resources.

## METHODS

This review was conducted in accordance with the Preferred Reporting Items for Systematic Reviews and Meta-Analyses 2020 guidelines.^29^ We systematically reviewed all abstracts published between 2018–2022 in PubMed and Scopus. Papers were screened by titles and/or abstracts by two blinded, independent researchers and co-authors. Conflicts were resolved by consensus with the principal investigator. Search terms included the following: prehospital or EMS or emergency medical services or ambulance or retrieval, vital sign, waveform measure, vital metric, blood pressure, pulse rate, heart rate, respiratory rate, SpO_2_ pulse oximetry, oximetry, plethysmograph, electrocardiogram, ECG, arterial pressure, pulse pressure, systolic, diastolic, electroencephalogram, EEG, end tidal carbon dioxide, ETCO_2_, waveform capnography, temperature, thermometer, heart rate variability, time domain, frequency domain, SDNN, continuous waveform measure, HF, LF, HF/LF, cerebral oximetry, cerebral tissue oximetry, near infrared spectroscopy, NIRS, rSO_2_, impedance, ohms, electrical impedance. We identified additional studies by reviewing references cited. For repeated cohorts, data are reported from the article with the latest publication date. Continuous variables are reported as mean +/− standard deviation for parametric variables and medians with interquartile range for non-parametric variables. Categorical variables are reported as percentages.

Inclusion criteria were studies published in English from 2018–2022 of any design that proposed or tested a novel patient monitoring system in prehospital, hospital, or resource-limited settings. The population included adults (≥ 18 years of age) in transit to the hospital or in the emergency department (ED). Interventions assessed were the patient monitoring systems themselves, and the primary endpoint of the study was measurement accuracy (defined as the agreement between experimental patient monitoring system-derived outputs and a validated reference platform). Secondary endpoints included clinical utility (eg, impact on decision-making and time to diagnosis or intervention), predictive capacity, feasibility (eg, ease of use, interference with workflow), and prototype design. We excluded studies if they lacked original data, described theoretical systems without evaluation of practicality, lacked essential components of scientific writing, or were not applicable to prehospital settings.

**Figure 1:**
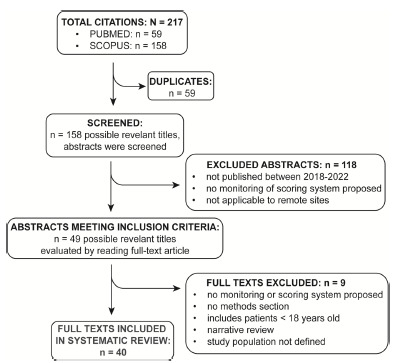
Flow diagram that summarizes the process by which studies were identified, screened, and ultimately selected for inclusion in this systematic review. *PRISMA*, Preferred Reporting Items for Systematic Reviews and Meta-Analyses.

**Figure 2:**
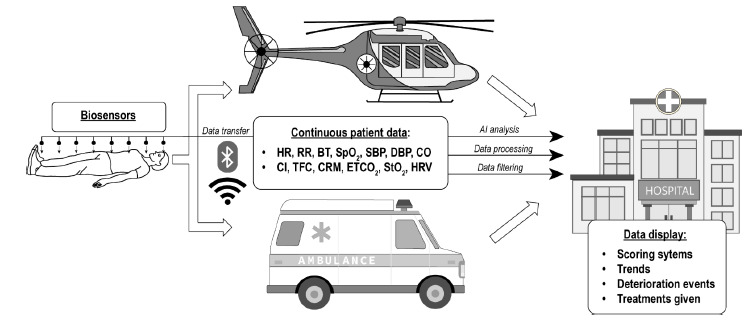
Overview of key concepts in remote patient monitoring systems with applicability in prehospital and resource-limited settings. *HR*, heart rate; *RR* respiratory rate; *SpO_2_*, oxygen saturation; *SBP*, systolic blood pressure; *DBP*, diastolic blood pressure; *CO*, cardiac output; *CI*, cardiac index; *HRV*, heart rate variability; *BT*, body temperature; *StO_2_*, tissue oximetry; *TFC*, thoracic fluid content, *CRM*, compensatory reserve metric; ETCO_2_, end-tidal carbon dioxide.

The above search criteria produced 59 and 158 hits from PubMed and Scopus online databases, respectively. After the removal of 59 duplicates, we screened 158 abstracts and/or titles for relevance to the proposed objective. Of the 158 abstracts and/or titles, 118 were excluded. The remaining 49 were read in their entirety, with 40 of them meeting inclusion criteria and included in the final review. The flow diagram of the study selection process is shown in Figure 1. A visual summary of the discussed concepts is shown in Figure 2.

## RESULTS

### Validation

Understanding the quality of the data produced by novel patient monitoring systems is essential before they can used to guide clinical decision-making. Validation studies evaluate whether experimental patient monitoring systems produce accurate data by comparing their results to trusted clinical benchmarks. Four studies assessed the accuracy of contact-dependent vital signs monitoring platforms. Glasin et al (2018) found good correspondence between vital sign measurements taken by the RespiHeart platform, a photoplethysmographic (PPG)-based sensor placed on the sternum, and the Phillips IntelliVue MP30 standard bedside monitor in 50 ED patients. However, the PPG-based sternal sensor was significantly slower at detecting rapid changes in RR.^30^ Tayfur and Afacam (2019) found good correspondence with standard monitors for HR and SpO_2_ measured by the Samsung Galaxy S8 in 101 ED patients.^31^ The compact, multiparameter handheld vital sign-monitoring device, the PICO monitor, was tested in 226 ED patients and showed high correspondence for all variables except temperature, which was consistently lower than the standard by 0.3 °C.^32^ In 2020, Sheridan et al introduced the Flowsense platform, a device that is intended to standardize capillary refill measurements across different users; however, its utility remains untested in prehospital settings.^33^

Four studies investigated the accuracy of contactless methods of vital sign monitoring. Zeng et al (2020) used ultra-wideband microwave imaging to estimate HR and RR. Here, accuracy was increased by processing more slow-time signals; however, simultaneous detection of both vital signs simultaneously remained poor.^34^ In 2022, Capraro et al used video PPG-motion analysis to approximate HR and RR in 475 ED patients, observing moderate agreement but with the requirement for exposed skin of the face and upper torso.^35^ Takahashi et al (2021) and Achermann et al (2019) used deep learning algorithms to estimate RR from thermal and video imaging data. In the former, a mean error of 0.66 breaths per minute was observed in seven healthy subjects; however, the platform could not detect apneic events.^36^ In the latter, camera-based prototype application-based detection achieved high sensitivity and specificity for the detection of tachypnea but required 60 seconds of continuous measurements and exposed skin.^37^ The main outcomes of studies validating patient monitoring systems are summarized in Table 1.

### Predictive Capacity

Platforms that accurately predict patient outcomes have important implications for early triage, risk-stratification, and identifying the need for lifesaving and often invasive interventions. Fourteen observational studies investigated the predictive capacity of various patient monitoring systems. Four studies used artificial intelligence (AI) to predict clinical outcomes. In 2018 Kim et al compared the revised trauma score to three machine-learning algorithms applied to a dataset of 460,865 trauma patients. The revised trauma score, a verified scoring system for trauma survival, includes systolic BP, Glasgow Coma Score (GCS), and RR. The study also introduced a simplified consciousness score, calculated automatically via a wearable monitor, as an alternative to the GCS. The machine-learning algorithms using simplified consciousness score and vital signs had the highest predictive power for survival (area under the receiver operating characteristic curve [AUROC] = 0.89; 95% CI, 0.882–0.890), outperforming the revised trauma score (AUROC = 0.78).^38^ A similar 2020 study showed that AI algorithms could make triage classifications of patients using continuous vital sign measurements with high fidelity.^39^

Paydar et al (2020) compared the accuracy of five AI modeling systems to predict prognosis in trauma patients after resuscitation within the first 24 hours after admission based on various clinical and paraclinical factors. Levels 1 and 2 blunt trauma patients were classified as critically ill or non-critically ill based on intensive care unit (ICU) admission, death, and need for emergency surgery. The AI systems were then retrospectively applied to the dataset, and the support vector machine and bagging algorithms classified patients as critical or non-critical with 99% precision. Clinical and paraclinical factors after resuscitation were also ranked by order of importance to the algorithm: GCS; hematocrit, diastolic BP; base excess; pH; SpO_2_; and bicarbonate. Of note, all these parameters except for diastolic BP were statistically different between the two patient groups prior to resuscitation.^40^ To predict patient decompensation at varying rates of resuscitation, Gupta et al (2022) trained a gradient-boosted regression tree machine-learning algorithm on arterial waveform patterns collected from 13 subjects during a simulated model of hemorrhage resuscitation. The authors found that training the algorithm on a single parameter, the half-rise to dicrotic notch, achieved a root mean square error of 13%, an *R*^2^ of 0.82, and AUROC of 0.97 for detecting decompensation.^41^

Two studies tested the predictive capacity of new scoring systems. Viglino et al (2020) developed the Early Warning Score O_2_ (EWS.O_2_) based on vital signs derived from continuous non-invasive monitoring. In 1,729 patients presenting to the ED with chief complaint of dyspnea, the EWS.O_2_ score predicted a composite outcome of non-invasive ventilation, ICU admission, and death with an AUROC of 0.704 (95% CI, 0.672–0.736); its predictive capacity was comparable to that of the SpO_2_/FiO_2_ ratio (AUROC = 0.695, *P* = .46) and increased vs the New Early Warning Score (NEWS) (AUROC 0.662, *P* < .01) and NEWS2 (AUROC = 0.672, *P* = .02) scores.^42^ In 2019, Prabhakar et al found that combining heart rate variability (with the quick Sequential Organ Failure Assessment (qSOFA) score produced a greater c-statistic than qSOFA alone for the prediction of 30-day mortality in 343 septic ED patients.^43^

Six studies evaluated the predictive capacity of various physiologic parameters that can be measured non-invasively and continuously, including cardiac output, cardiac index, thoracic fluid content, compensatory reserve metric (CRM, retrospectively determined from the PPG waveform algorithmically), pulse character (estimated as SBP < 100), SpO_2_, ETCO_2_, SBP, tissue oximetry, and heart rate variability (specifically the low-frequency/high-frequency [LF/HF] ratio). Gho et al (2021) used electrical cardiometry to monitor thoracic fluid content of 368 pneumonia patients with a presenting chief complaint of dyspnea; the AUROCs for 28-day mortality and ICU admission were 0.72 (95% CI, 0.71–0.74) and 0.73 (95% CI, 0.62–0.82), respectively.^44^

Javaudin et al (2018) evaluated the capacity of SpO_2_, ETCO_2_, and systolic BP to predict 30-day neurologic outcomes in patients who arrested and achieved return of spontaneous circulation (ROSC) in the prehospital setting; significant relative risks of worse outcomes were observed for SpO_2_ < 94% (RR 1.108, 95% CI, 1.069–1.147), ETCO_2_ < 30 or > 40 (< 20 RR 1.191 (95% CI, 1.143–1.229); 20–29, RR 1.092 (95% CI, 1.061–1.123); 41–50, RR 1.075 (95% CI, 1.039–1.110); > 50, RR 1.136 (1.085–1.179), and SBP < 100 or > 130 mm Hg.^18^ In 300 trauma patients, the combination of CRM and PC predicted need for life-saving intervention (defined as need for transfusion, intubation tube thoracostomy, or operative/angiographic hemorrhage control) with an odds ratio (OR) of 9.91 (95% CI, 4.08–24.09; *P* < .001).^45^

In another study, handheld tissue oximetry (StO_2_) was performed en route in 88 trauma patients transported to the hospital via helicopter; no clinically useful correlations were found between StO_2_ and occult hemorrhagic shock prediction (*r* = −0.17; 95% CI, −0.33–1.0, *P* = .94) or need for life-saving interventions (OR 1.03, 95% CI, 0.96–1.1, *P* = .46).^46^ Chukwulebe et al (2021) showed that the admission rate from the ED was better predicted by serum lactate (AUROC = 0.83, 95% CI, 0.64–0.92) than cardiac output (AUROC = 0.59, 95% CI, 0.41–0.73) and cardiac index (AUROC = 0.63, 95% CI, 0.36–0.80) in 50 ED patients at risk for sepsis.^47^ In another prospective observation study of 466 patients presenting to the ED with signs of sepsis, the LF/HF of heart rate variability showed poor reliability as a clinical predictor of critical illness and death, both as a single variable and alongside SOFA scoring.^48^

Two studies investigated physiological changes during prehospital care. Walker et al (2018) analyzed continuous vital signs collected during paramedic-performed rapid sequence intubation in the field, noting a 95% intubation success rate, with desaturation events occurring primarily during the first intubation attempt.^49^ In a cohort of 477 traumatic brain injury patients transported by air, Davis et al (2022) found no correlation between hemodynamic events and phases of air transport.^50^ The key outcomes of these predictive capacity studies are summarized in Table 2.

### Clinical Utility and Feasibility

For the purposes of this review, clinical utility refers to the ability of a patient monitoring system to generate timely, actionable data with the potential to inform decision-making and impact outcomes, while feasibility reflects the system’s ease of use and its ability to integrate into existing workflows without impeding care. Nine papers studied the clinical utility and feasibility of various patient monitoring systems in prehospital and hospital settings, including one randomized clinical trial (RCT) and eight observational studies. Reed et al (2018) evaluated the utility of continuous smartphone-based event recording for the timely diagnosis of symptomatic arrythmias not initially captured upon initial presentation to the ED in patients with palpitations and presyncope. In this RCT of 243 patients, the smartphone app achieved a 90-day detection rate of 55.6% compared to 9.5% in the control group discharged without the app (*P* < .001).^51^

Hansen et al (2019) showed that continuous non-invasive arterial pressure monitoring was feasible to employ during emergency scenarios, delivering accurate readings in prehospital settings with no adverse events or obstruction of normal emergency care protocols.^52^ A subsequent study published in 2020 proposed a more comprehensive system that combined continuous non-invasive vital-sign monitoring with an integrated “e-triage” system, allowing for quick determination of patient priority status and immediate data transmission to receiving hospitals via Bluetooth connectivity. The prototype was presented to 30 emergency physicians and EMS personnel and evaluated as per the technology acceptance model; it was found that the path coefficients between perceived usefulness and rural environment, urban environment, patient status, and behavioral intention displayed statistical significance.^53^

Poncette et al (2022) determined that the utility of a patient monitoring system could be improved by implementing human-centered design approaches. Here, the authors tested the prototype before (prototype A) and after (prototype B) incorporating changes in accordance with feedback from five ICU attendings. They found that, through modification of the prototype with user-based feedback (particularly feedback involving the user interface), perceived usability (mean[A] = 68.5, mean[B] = 89, *P* = .03), performance efficiency (normative path deviation [NPD] mean[A] = 8.8, NPD mean[B] = 3.2, *P* = .01) and effectiveness (task completion rate [TCR] A = 61%, TCR[B] = 100%) were significantly increased.^54^ The importance of a streamlined, user-friendly interface was redemonstrated in a 2022 study of Israeli military rescue operations. In that study, a low-profile wearable patient monitoring system device (the Bladeshield 101) with a digital user interface was shown to significantly increase documentation of vital signs and life-saving interventions in 221 combat casualties compared to standard paper casualty cards requiring handwritten recordkeeping.^55^

Three studies evaluated the feasibility and utility of implementing various patient monitoring systems to improve care for critical trauma and cardiac arrest patients. Two observational studies from 2019 and 2020 evaluated the feasibility of implementing non-invasive cardiac output and cerebral blood oxygenation monitoring devices in emergent settings (specifically Level 1 trauma and cardiac arrest patients, respectively). Kuster et al (2019) trialed the ICON non-invasive cardiac output monitoring (NICOM) device in 20 Level 1 trauma patients following transfer to the ED. Application of the device involved placement of four small electrodes on the skin across the left hemithorax. The authors found no observable adverse effects on standard-of-care ED practices and minimal disruptions in continuous signal transmission over 60 minutes of monitoring.^56^

Yagi et al (2020) evaluated the feasibility of employing the low-profile and easily transportable near-infrared spectroscopy device for real-time cerebral blood oxygenation monitoring in cardiac arrest patients actively undergoing resuscitative measures; the authors found no obstruction to standard-of-care practices and noted consistent synchrony between chest compressions and waveforms in a small cohort of 20 patients. Notably, due to the device’s small size, it was amenable to use in the prehospital setting including ambulance and air transport.^57^ Zanatta et al (2020) showed that ultrasound could be used to improve cardiopulmonary resuscitation quality in real time by guiding hand placement and compression depths that maximize cardiac squeeze.^58^

While most studies in this subsection focused on continuous monitoring platforms, Kim and Jin (2022) sought to maximize the utility of a fixed number of data points by determining the optimal temporal distribution of registered nurse (RN)-mediated vital sign checks. They performed a cross-sectional study on 25,751 monitored adult ED visits over a year. To compare different charting strategies objectively, they described two separate quantities: coverage and capture. Coverage was defined as the proportion of monitor-derived vital sign measurements that fell within the bounds of RN-charted values, and capture was defined as the documentation of a vital sign abnormality (ie, HR > 100 or < 60, mean arterial pressure < 65, and SpO_2_ < 95) detected by bedside monitor. Across all the charting strategies evaluated, capture and coverage were significantly increased with strategies that contained an increased frequency of charting events toward the start of the encounter, ultimately increasing deterioration events in a timely fashion.^59^ Although this study was not performed in prehospital settings, it is reasonable to speculate that increasing vital sign charting at an earlier time point (ie, first patient contact) would yield more pronounced benefits; the outcomes of studies evaluating the utility and feasibility of patient monitoring systems are summarized in Table 3.

### Design

Nine descriptive studies described the design of a theoretical patient monitoring system for use in remote settings. Each prototype contained three general components that facilitated the flow of patient data: biosensors, interfaces, and communications systems. Two studies focused on the design of a novel composite biosensor prototype. Phan et al (2022) proposed a wearable biosensor patch that continuously measures body temperature, BP, and ECG tracings. In addition to biosensors, the patch was embedded with a microcontroller, GPS, and Bluetooth module. Temperature and ECGs were measured directly, and BP was estimated through its correlation with the pulse arrival time (determined with AI processing of PPG and ECG data). The patch was tested on five healthy subjects, and the BP estimation algorithm displayed high correlations for SBP and DBP prediction (*R* = 0.86 and 0.84, respectively).^60^ Walinjkar (2018) proposed a similar prototype but expanded upon the use of filters and algorithms to increase the accuracy of vital sign measurements. The author proposed an additional feature of determining the shortest path to the nearest hospital through network analysis of GPS coordinates.^61^

The remaining seven studies focused on systems of data integration and transmission to receiving hospitals. Various biosensors, microcontrollers, and communications systems were proposed to relay continuous vital sign measurements to receiving hospitals. While most systems included monitoring of vital signs only (ie, BP, SpO_2_, body temperature, HR, and RR), additional features were included in some studies. Notably, Naregalkar and Krishna (2019) proposed an ambulance-based, vital sign monitoring system, which included a portable camera for real-time video-monitoring, spirometry for lung function analysis, and a handheld dynamometer to assess muscle fatigue.^62^ Zainuddin et al (2020) used portable cameras along with deep-learning algorithms to determine the real-time emotional status of patients with seven identifiable emotional states.^63^ Nagayo et al (2021) described a remote patient monitoring system equipped with a drone capable of delivering a 500-gram medical kit across a football field,^64^ and Billis et al (2019) included additional discussions about the utility of intelligent bio-monitoring sensors and AI algorithms to stratify parents based on the acuity level of care required.65 Components of the proposed patient monitoring systems are included in Table 4.

## DISCUSSION

Consistent with prior reviews, limited data and lower levels of evidence currently preclude the creation of evidence-based guidelines.^66^ Many devices remain in validation phases, and no biomarker or novel vital sign has convincingly shaped outcomes. Moreover, cost remains a key limitation across nearly all technologies described. Many proposed systems incorporate advanced biosensors, proprietary components, or cellular services that may be unaffordable or unsustainable in resource-limited settings. Even devices that are relatively inexpensive at baseline often require regular maintenance, calibration, or technical support, which may exceed the capacity of under-resourced health systems. Therefore, financial and operational sustainability represent significant barriers to widespread implementation. Despite this, many promising platforms are currently under investigation and may lead to more rigorous studies, including RCTs. We excluded from this review surgically implantable sensors, which are less applicable to emergency triage and are discussed elsewhere.^67^

For devices in validation phases, several practical and contextual limitations warrant further discussion. Multiparameter, handheld, vital sign monitoring devices, which record vital signs and ECG in real time, require seven contact points, limiting scalability in mass casualty scenarios. While PPG-based sternal sensors require only a single contact point and allow for rapid triage, they provide limited physiologic data and depend on an external display device for interpretation. Smartphone-based systems, while inexpensive and widely available, require precise finger positioning, limiting their practicality in remote triage scenarios with low clinician-patient ratios.^68^ Moreover, most studies in validation phases were conducted in idealized or hospital-based settings, not field settings, and often with healthy patients. Thus, generalizability to settings where cost, environmental factors, low staff-to-patient ratios, and poor healthcare infrastructure, is limited.

Other patient factors (eg, hemodynamics, comorbidities), transport conditions (eg, terrain, altitude), and logistical challenges (eg, device setup) remained underexplored. Further research is needed to test these devices in prehospital and remote conditions. Technical barriers also persist; these include unreliable skin-based temperature readings and failure to detect low apnea, a key predictor of clinical deterioration.^66^,^69^,^70^ Considering the crucial role of BP monitoring in hemodynamics and resuscitation, its absence limits its use in emergency response.

The COVID-19 pandemic has driven interest in contactless vital sign monitoring platforms.^71^ However, these systems often require increased equipment and processing power, which may make them more suitable for isolation rooms in resource-rich hospitals rather than remote triage. Moreover, prehospital triage typically involves direct patient contact, often within confined spaces such as ambulances, helicopters, or planes. Studies found no disruption to standard care practices with integration of these platforms. Worker efficiency and satisfaction were increased by streamlining protocols, and platforms were well-received by emergency and hospital personnel. Notably, CNAP showed good accuracy for BP measurement in the field and during transport, although it was unreliable in patients with SBP < 90 mm Hg, raising concerns about its reliability in hemorrhagic trauma.^52^, ^72^

Smart health platforms, which combine machine learning-driven AI technologies with the Internet of Things-enabled medical devices, offer potential for remote triage by providing real-time data and personalized predictions.^73^ However, these systems require training on large datasets, and further validation will be needed to assess their performance in diverse patient populations under non-ideal conditions. Cost-benefit analyses for these platforms are under investigation.^74^ Moreover, while these platforms hold potential for chronic disease monitoring, their role in triage remains unclear.^75^

Driven by advancements in the Internet of Things, recent trends in patient monitoring system research have shifted toward specific applications and system architecture.^76^ The increasing accessibility of instilling devices with internet connectivity leads to heterogenous networks that are largely unregulated from the standpoints of quality and security.^77^ Many of these studies were conducted out of necessity in regions with limited internet access; however, dead zones are becoming increasingly scarce across the US.^78^ Nevertheless, these studies describe the various components that can be used to design monitoring systems with specific, customizable purposes. However, to ensure reliable data transfer of protected health information, continued centralization and standardization of these platforms are essential as technology advances.^79^

## LIMITATIONS

Many included studies were conducted in controlled settings and healthy participants, thus greatly limiting external validity. Study designs were very heterogeneous, and there is a lack of standardized outcomes measures. Further, the wide range of technical variabilities limited our ability to synthesize and group data accordingly. There is also potential publication bias, as research on techniques that did not produce positive outcomes was likely not published. Our review may also be limited by the scope of the literature search, which included only two English-language databases and did not involve contacting authors. Therefore, relevant unpublished data or studies indexed elsewhere may have been missed. In addition, because of the considerable heterogeneity in study designs, outcome measures, and the early-stage nature of many technologies, we were unable to perform formal risk-of-bias assessments or evidence grading. Further high-quality research, including RCTs, is needed before the data can be meaningfully synthesized or used to support clinical guidelines. The available literature is not mature enough to support broad clinical recommendations.

## CONCLUSION

Many remote patient-monitoring platforms are progressing beyond validation but remain in early stages of clinical utility evaluation. Rigorous randomized studies are needed to assess their impact on outcomes. Cost-benefit analyses during prehospital transport are notably lacking but are essential for guiding adoption in clinical settings.

## Figures and Tables

**Table 1:** Summaries of eight studies that validate the accuracy of patient monitoring systems, including those providing continuous or intermittent vital sign measurements.

Study	Primary outcome(s)	Main finding(s)
(Glasin et al, 2018)	Agreement between RespiHeart- and reference-generated HR, RR, and SpO_2_ measurements	The RespiHeart is a wearable, continuous vital sign-monitoring device that uses dual wavelength PPG to detect HR, RR, and SpO_2_; measurements produced with the RespiHeart are in good agreement with those derived from reference monitors.
(Tayfur and Afacan, 2019)	Agreement between smartphone- and reference-generated HR and SpO_2_ measurements	HR and SpO_2_ obtained via the Samsung Health app on the Samsung Galaxy S8 and showed excellent correlation with reference measurements.
(Takahashi et al, 2021)	Agreement between thermography- and reference-generated RR measurements	Contactless RR approximations can be made using thermal imaging of the face and deep machined learning data analysis via YOLO v3, with a mean absolute error of 0.66 breaths per minute versus reference measurements
(Capraro et al, 2022)	Agreement of HR and RR measurements made via vPPG and PIT devices, respectively, and reference measurements	Contactless vPPG and PIT-derived HR and RR measurements demonstrated good agreement with contact-based reference measurements in a live ED triage setting.
(Zeng et al, 2020)	Agreement between UWB biosensor- and reference-generated RR measurements	A low-profile, low-cost UWB biosensor is designed and tested; by using microwaves to measure the millimeter displacement of the chest wall, the device can accurately detect HR and RR in real time.
(Achermann et al, 2019)	Sensitivity and specificity to detect tachypnea (RR > 20)	Tachypnea can be accurately detected using contactless CBPA with test sensitivity and specificity of 97.4% and 87.8%, respectively.
(Sheridan et al, 2020)	N/A	The Flowsense is a capillary refill-measuring device that attempts to standardize capillary refill assessment across users and may assist in the early diagnosis of sepsis.
(Renier et al., 2019)	Analytical accuracy of PICO monitor-generated SpO_2_, HR, RR, T, and 7-lead ECG tracings	The handheld PICO monitor is a wireless and lightweight monitoring device equipped with Bluetooth capabilities that accurately measures SpO_2_ HR, RR, T, and 7-lead ECG tracings in real time.

*HR*, heart rate; *RR*, respiratory rate; *SpO_2_*, oxygen saturation; *PPG*, photoplethysmography; *vPPG*, video photoplethysmography; *PIT*, passive infrared thermometry; *ED*, emergency department; *UWB*, ultra-wideband; *CBPA*, camera-based prototype application; *T*, temperature; *ECG*, electrocardiography.

**Table 2:** Summaries of 14 studies that assessed the predictive capacity of patient monitoring systems, novel scoring tools, and other quantifiable physiologic parameters to predict clinical outcomes, including mortality, intensive care unit admission, clinical decompensation, and the need for life-saving interventions.

Research study	Primary outcome(s)	Main finding(s)
(Viglino et al, 2020)	Poor outcome	The EWS.O_2_ score, a new automatable monitoring tool incorporating RR, HR, SpO_2_, and FiO_2_, predicts poor outcome with increased predictive accuracy compared to NEWS and NEWS2 scoring systems in patients presenting to the ED with a chief complaint of dyspnea.
(Chukwulebe et al, 2021)	Hospital admission	Serial non-invasive hemodynamic monitoring of CO, CI, SV, and HR (as measured via the NICOM has inferior capacity to predict hospital admission) in ED patients with 2 of 3 SIRS criteria compared to serum lactate.
(Paydar et al, 2021)	Critical illness	The Bagging and SVM methods of CRISP-DM could predict the development of critical illness in trauma patients after resuscitation with 99% accuracy using GCS, base excess, and SBP as the most-fitted variables.
(Gupta et al, 2022)	Hemodynamic decompensation	The half-rise to dicrotic notch, an arterial blood pressure waveform measured non-invasively via the Finapres technology Finometer, can be used to train a gradient-boosted regression machine-learning algorithm to accurately detect decompensation in a simulated, lower body pressure model of hemorrhage and whole blood resuscitation.
(Davis et al, 2022)	Hemodynamic events for each phase of flight experienced by critically injured combat casualties with TBIs transported by plane	No significant correlation was found between hemodynamic events and phase of flight in critically injured combat casualties with TBIs transported by plane.
(Ciaraglia et al, 2022)	Blood transfusion within 24 hours of triage	Tachypnea can be accurately detected using contactless CBPA with test sensitivity and specificity of 97.4% and 87.8%, respectively.
Life-saving intervention	N/A	The Flowsense is a capillary refill-measuring device that attempts to standardize capillary refill assessment across users and may assist in the early diagnosis of sepsis.
Composite outcome	The combination of abnormal pulse character (defined as SBP < 100 mm Hg and measured in the prehospital setting) with abnormal CRM (defined as CRM < 60% and measured upon arrival to the hospital) significantly increased predictive capacity for clinical outcomes vs either parameter alone.	The handheld PICO monitor is a wireless and lightweight monitoring device equipped with Bluetooth capabilities that accurately measures SpO_2_, HR, RR, T, and 7-lead ECG tracings in real time.
(Gho et al, 2021)	Mortality within 28 days of initial presentation to ED	Thoracic fluid content, as measured via a portable and noninvasive electrical cardiometry monitoring device, accurately predicted mortality of patients with PNA at 28 days after initial ED visit.
(Naemi et al, 2020)	Clinical severity of patient condition	Trained on continuously monitored and individualized patient vital signs (HR, RR, SpO_2_, and SBP), the LSTM neural network more accurately predicted real-time fluctuations in illness severity compared to the MLP neural network.
(Radowsky et al, 2019)	Occult shock	Tissue oxygenation, as measured via a handheld near-infrared spectroscopy-based oximeter, showed no significant predictive capacity to identify occult shock in trauma patients requiring air ambulance transport.
(Prabhakar et al., 2019)	All-cause 30-day mortality	The predictive capacity of the qSOFA score to distinguish sepsis survivors vs non-survivors is enhanced by the addition of detrended fluctuation analysis α2, a heart rate variability assessed by electrocardiography monitoring.
(Javaudin et al., 2018)	Patient neurological status 30 days after initial cardiac arrest	In patients who suffered cardiac arrest and achieved ROSC in the field, prehospital vital parameters including SpO_2_ ≥ 94%, ETCO_2_ of 30−40 mm Hg, and SBP of 100–130 mm Hg were associated with better neurological status 30 days post-arrest.
(Walker et al., 2018)	Recorded survival after discharge into the ED	Injury survivability can be accurately predicted by machine-learning algorithms trained on variables that are easily measured in the prehospital setting (including age, SBP, HR, RR, and consciousness score); these predictions have special applicability to mass casualty scenarios when resources are severely limited.
(Barnaby et al, 2018)	Alterations in SpO_2_, HR, and SBP during prehospital RSI	Most physiological alterations associated with prehospital RSI occurred during the first attempt, which was successful in 82% of cases.
(KIm et al, 2018)	Need for endotracheal intubation Need for NIVS for ≥ 1 hour HD support for ≥ 1 hour ICU admission with LOS ≥ 24 hours Death within 72 hours after presentation	The low-frequency/high-frequency ratio of HRV was 34% sensitive in identifying patients who required ICU admission or died within 72 hours from time of presentation; its capacity to predict short-term clinical deterioration was not significantly augmented by the inclusion of the qSOFA score.

*EWS*, Early Warning Score; *HR*, heart rate*; RR*, respiratory rate; *SpO_2_*, oxygen saturation; *FiO_2_*, oxygen flow rate derivative*; NICOM*, non-invasive cardiac output monitoring; *ED*, emergency department*; SIRS*, systemic inflammatory response syndrome; *SVM*, support vector machine*; CRISP-DM*, cross-industry standard process for data mining; *GCS*, Glasgow Coma Scale; *SBP*, systolic blood pressure; *CRM*, compensatory reserve metric; *TBI*, traumatic brain injury; *PNA*, pneumonia; *LSTM*, long short-term memory*; ROSC*, return of spontaneous circulation; *ETCO_2_*, end-tidal carbon dioxide*; RSI*, rapid-sequence intubation; *qSOFA*, quick sequential organ failure assessment; *ICU*, intensive care unit; *HRV*, heart rate variability.

**Table 3:** Summary of nine studies evaluating the utility and feasibility of patient monitoring systems in emergency and prehospital settings and results demonstrating that these platforms are generally feasible to implement, non-obstructive to typical hospital workflows, and associated with improved documentation, clinician workflow, and early detection of clinical deterioration.

Study	Primary outcome(s)	Main finding(s)
(Kim and Jin, 2022)	Coverage Capture	The prompt recognition of clinical deterioration from intermittent vital sign documentation is improved by specific strategies without increasing overall workload.
(Koceska et al, 2020)	Perceived usefulness/ease of use Attitude toward usage Intention to use the system Patient status Environment	Paramedics and emergency physicians perceive a mobile monitoring system that uses non-intrusive wireless sensors to continuously measure vital parameters as useful in both urban and rural environments.
(Poncette et al, 2022)	Effectiveness Efficiency Perceived usability	Patient safety in hospital settings is improved by the continuous monitoring of vital signs, and technological platforms developed with basic human-centered design methods and principles have a higher likelihood to positively affect clinical decision-making.
(Kuster et al, 2019)	Device safety Device reliability Device user-friendliness	Continuous non-invasive cardiac output monitoring via the NICOM device is feasible and safe for the initial hemodynamic evaluation of trauma patients and can be implemented without interfering with standard trauma patient protocols.
(Yagi et al, 2020)	Synchrony of chest compression to cerebral blood flow waveform TOI following CPB NIRO-CCR1 pulse rate (tempo) detection	The quality of CPR may be improved using the NIRO-CCR1, a small and easy-to-transport device that can detect pulse rate (CPR tempo) and monitor CBO in real time.
(Reed et al., 2019)	Symptomatic rhythm detection at 90 days	The use of personal cell-phone event recorders significantly increased the rate of detection of symptomatic cardiac rhythms at 90 days in patients presenting to the ED with palpitations and normal ECGs.
(Sorkin et al, 2022)	Time from injury to transfer of data to trauma registry Documentation of vital signs, timing, and treatment provided	The BladeShield 101 digital wearable combat card provides continuous vital sign monitoring and increases the completion percentage of medical record keeping compared to standard paper casualty cards.
(Zanatta et al. 2020)	Ability to assess and improve CPR quality in real-time via POCUS Thorax location that produces the best hemodynamic effect of CPR	POCUS can be used to improve the quality of prehospital CPR in real time by guiding hand placement to the location on the thorax that maximizes left ventricular squeeze.
(Hansen et al, 2019)	Feasibility of CNAP use in prehospital settings	The CNAP is a feasible method of prehospital BP monitoring requiring only 30 minutes of training and providing continuous readings after a median of 164.5 seconds.

*EM*, emergency medicine; *NICOM*, non-invasive cardiac output monitoring; *CPR*, cardiopulmonary resuscitation; *CBP*, cardiopulmonary bypass; *TOI*, tissue oxygenation index; *ED*, emergency department; *POCUS*, point-of-care ultrasound; *CNAP*, continuous noninvasive arterial pressure; *BP*, blood pressure; *CBO*, cerebral blood oxygenation.

**Table 4:** Summary of nine descriptive studies that proposed theoretical patient monitoring systems tailored for use in rural and/or resource-limited prehospital environments.

Manuscript	Parameter(s)	Biosensor(s)	Microcontroller(s)	Device communication	Comments
(Phan et al, 2022)	BP ECGT T BM HR	ADS1293 MAX30205 BNO055/9-axis accelerometer	PIC16LF19186	CC2560 low-energy Bluetooth module PAM-7Q GPS antenna module Firebase cloud network IoT	Includes an SMS alert system that provides real-time updates on patient status
(Habib et al, 2022)	T SpO_2_ HR	DS18B20 MAX30102 AD8232	Arduino Uno	HC-05 (Bluetooth)	Uses an algorithm to calculate HR from ECG tracing
(Valdez et al, 2022)	T SpO_2_ HR	MAX30100 DS18B20	NodeMCU	IoT Cloud server	
(Merza and Qudra, 2022)	ECGT T HR	AD8232 Pulsesensor MAX6675	Arduino	IoT Sim800l cellular module 3G network Raspberry pi server	Includes an SMS alert system on doctor’s mobile phones
(Nagayo et al, 2021)	BP T SpO_2_ RR LOC	MAX30100 MLX90614 DS18B20 SUNROM 1437 Passive infrared motion sensor	Not specified	Not specified	Also confirmed the ability of the prototype drone to successfully carry a 500-gram medical kit across a football field
(Zainuddin et al, 2020)	T HR ES	Raspberry pi camera Heart rate sensor Thermal sensor	NodeMCU Raspberry pi	Thingsboard IoT cloud	Trained using FER2013 dataset
(Billis et al, 2019)	SpO_2_ HR RR Patient Location	HR sensor RR sensor SpO_2_ sensor Tracking device	Not specified	RESTful web services	Does not specify which sensors are used
(Naregalkar and Krishna, 2019)	ECGT BP HR T MF PF	Oscillometer 3-lead ECG Dynamometer Spirometry Hand-grip HR sensor Thermistor	NI USB-6281 Data Acquisition board	Internet Toolkit and Web Publishing Toolkit of LabVIEW 3G network	
(Walinjkar, 2018)	ECGT SpO_2_ BM RR SBP	AD8232 MAX30101 3-axis accelerometer	AM335x-based Beaglebone Black	NEO-6 series GPS module	Indirectly calculated, not directly measured

*BP*, blood pressure; *ECGT*, ECG tracing; *ES*, emotional state; *T*, temperature; *BM*, body movement; *HR*, heart rate; *IoT*, internet of things; *SpO_2_*, pulse oxygenation.

## References

[1] MacKenzie EJ, Rivara FP, Jurkovich GJ (2006). A national evaluation of the effect of trauma-center care on mortality. N Engl J Med.

[2] Celso B, Tepas J, Langland-Orban B (2006). A systematic review and meta-analysis comparing outcome of severely injured patients treated in trauma centers following the establishment of trauma systems. J Trauma.

[3] Newgard CD, Schmicker RH, Hedges JR (2011). A multisite assessment of the American College of Surgeons Committee on Trauma field triage decision scheme for identifying seriously injured children and adults. J Am Coll Surg.

[4] Tran A, Nguyen P, Mahmood I (2018). Permissive hypotension versus conventional resuscitation strategies in adult trauma patients with hemorrhagic shock: a systematic review and meta-analysis of randomized controlled trials. J Trauma Acute Care Surg.

[5] Newgard CD, Fu R, Bulger EM (2022). National guideline for the field triage of injured patients: recommendations of the National Expert Panel on Field Triage, 2021. J Trauma Acute Care Surg.

[6] Newgard CD, Gorman JD, Foresman BH (2010). A critical assessment of the out-of-hospital trauma triage guidelines for physiologic abnormality. J Trauma.

[7] Krieger AR, Ruzek E, Nasr I (2012). Efficacy of anatomic and physiologic indicators versus mechanism of injury criteria for trauma activation in pediatric emergencies. J Trauma Acute Care Surg.

[8] Barton CW, Hemphill JC, Morabito DJ (2005). A novel method of evaluating the impact of secondary brain insults on functional outcomes in traumatic brain-injured patients. Acad Emerg Med.

[9] Manley G, Knudson MM, Morabito D (2001). Hypotension, hypoxia, and head injury: frequency, duration, and consequences. Arch Surg.

[10] Spaite DW, Bobrow BJ, Keim SM (2022). Optimal out-of-hospital blood pressure in major traumatic brain injury: a challenge to the current understanding of hypotensio*n*. Ann Emerg Med.

[11] Spaite DW, Hu C, Bobrow BJ (2017). Association of out-of-hospital hypotension depth and duration with traumatic brain injury mortality. Ann Emerg Med.

[12] Spaite DW, Hu C, Bobrow BJ (2017). The effect of combined out-of-hospital hypotension and hypoxia on mortality in major traumatic brain injury. Ann Emerg Med.

[13] Spaite DW, Hu C, Bobrow BJ (2017). Mortality and prehospital blood pressure in patients with major traumatic brain injury: implications for the hypotension threshold. JAMA Surg.

[14] Spaite DW, Hu C, Bobrow BJ (2019). Association of statewide implementation of the prehospital traumatic brain injury treatment guidelines with patient survival following traumatic brain injury: the Excellence in Prehospital Injury Care (EPIC) study. JAMA Surg.

[15] Gaither JB, Spaite DW, Hu C (2021). Effect of implementing the out-of-hospital traumatic brain injury treatment guidelines: the Excellence in Prehospital Injury Care for Children study (EPIC4Kids). Ann Emerg Med.

[16] Bray JE, Stub D, Bloom JE (2014). The association between systolic blood pressure on arrival at hospital and outcome in adults surviving from out-of-hospital cardiac arrests of presumed cardiac aetiology. Resuscitation.

[17] Chiu YK, Lui CT, Tsui KL (2018). Impact of hypotension after return of spontaneous circulation on survival in patients of out-of-hospital cardiac arrest. Am J Emerg Med.

[18] Javaudin F, Lavaille L, Leclere B (2018). Impact of prehospital vital parameters on the neurological outcome of out-of-hospital cardiac arrest: results from the French National Cardiac Arrest Registry. Resuscitation.

[19] Smida T, Lavrentieva A, Giannakopoulos G (2022). Association of prehospital hypotension depth and dose with survival following out-of-hospital cardiac arrest. Resuscitation.

[20] Lacocque J, Siegel L, Sporer KA (2021). Prehospital, post-ROSC blood pressure and associated neurologic outcome. Am J Emerg Med.

[21] Smida T, Lavrentieva A, Giannakopoulos G (2024). The association of combined prehospital hypotension and hypoxia with outcomes following out-of-hospital cardiac arrest resuscitation. Prehosp Emerg Care.

[22] Smida T, Lavrentieva A, Giannakopoulos G (2022). Association of prehospital post-resuscitation peripheral oxygen saturation with survival following out-of-hospital cardiac arrest. Resuscitation.

[23] Chen WL, Liu CY, Yang SP (2009). Heart rate variability predicts short-term outcome for successfully resuscitated patients with out-of-hospital cardiac arres*t*. Resuscitation.

[24] Cooke WH, Salinas J, Convertino VA (2006). Heart rate variability and its association with mortality in prehospital trauma patients. J Trauma.

[25] Riordan WP, Copes WS, Shafi S (2009). Early loss of heart rate complexity predicts mortality regardless of mechanism, anatomic location, or severity of injury in 2178 trauma patients. J Surg Res.

[26] Ryan ML, Thorson CM, Otero CA (2011). Heart rate variability is an independent predictor of morbidity and mortality in hemodynamically stable trauma patient*s*. J Trauma.

[27] Liu NT, Holcomb JB, Wade CE (2014). Utility of vital signs, heart rate variability and complexity, and machine learning for identifying the need for lifesaving interventions in trauma patients. Shock.

[28] Liu NT, Holcomb JB, Wade CE (2015). Improving the prediction of mortality and the need for life-saving interventions in trauma patients using standard vital signs with heart-rate variability and complexity. Shock.

[29] Page MJ, McKenzie JE, Bossuyt PM (2021). The PRISMA 2020 statement: an updated guideline for reporting systematic reviews. BMJ.

[30] Glasin J, Henricson J, Lindberg LG (2018). Wireless vitals—proof of concept for wireless patient monitoring in an emergency department setting. J Biophotonics.

[31] Tayfur I, Afacan MA (2019). Reliability of smartphone measurements of vital parameters: a prospective study using a reference method. Am J Emerg Med.

[32] Renier WS, Lammers RL, Padeken D (2019). Analytical accuracy of the handheld PICO monitoring device during emergencies. BMJ Innov.

[33] Sheridan DC, Spiro DM, Hemphill NM (2020). Cutting-edge technology for rapid bedside assessment of capillary refill time for early diagnosis and resuscitation of sepsis. Front Med (Lausanne).

[34] Zeng X, Zhang Y, Huang Y (2020). Investigation of an ultra wideband noise sensor for health monitoring. Sensors (Basel).

[35] Capraro GA, Balmaekers B, Den Brinker AC (2022). Contactless vital signs acquisition using video photoplethysmography, motion analysis and passive infrared thermography devices during emergency department walk-in triage in pandemic conditions. J Emerg Med.

[36] Takahashi Y, Gu Y, Nakada T (2021). Estimation of respiratory rate from thermography using respiratory likelihood index. Sensors (Basel).

[37] Achermann S, Greif R, Wirthmüller U (2019). Contact-free monitoring of respiratory rates for triage of patients presenting to the emergency department. Resuscitation.

[38] Kim D, Jin BT, Song KJ (2018). A data-driven artificial intelligence model for remote triage in the prehospital environment. PLoS One.

[39] Naemi A, Najafi B, Boulton AJM (2020). Personalized predictive models for identifying clinical deterioration using LSTM in emergency departments. Stud Health Technol Inform.

[40] Paydar S, Abounoori M, Dehbozorgi A (2021). Do clinical and paraclinical findings have the power to predict critical conditions of injured patients after traumatic injury resuscitation? Using data mining artificial intelligence. Chin J Traumatol.

[41] Gupta JF, Rathore A, Chatterjee R (2022). Noninvasive monitoring of simulated hemorrhage and whole blood resuscitation. Biosensors (Basel).

[42] Viglino D, L’her E, Maltais F (2020). Evaluation of a new respiratory monitoring tool “Early Warning ScoreO_2_” for patients admitted at the emergency department with dyspnea. Resuscitation.

[43] Prabhakar SM, Thomas M, George J (2019). Combining quick sequential organ failure assessment score with heart rate variability may improve predictive ability for mortality in septic patients at the emergency department. PLoS One.

[44] Gho K, Yoon SY, Kim HJ (2021). Predictive and prognostic roles of electrical cardiometry in noninvasive assessments of community-acquired pneumonia patients with dyspnea. Hong Kong J Emerg Med.

[45] Ciaraglia AV, de la Fuente M, Baez AA (2022). Compensatory reserve and pulse character: enhanced potential to predict urgency for transfusion and other life-saving interventions after traumatic injury. Transfusion.

[46] Radowsky JS, Frakes MA, Thomas SH (2019). Handheld tissue oximetry for the prehospital detection of shock and need for lifesaving interventions: technology in search of an indication?. Air Med J.

[47] Chukwulebe SB, Walsh BT, Isaacson AE (2021). Early hemodynamic assessment using NICOM in patients at risk of developing sepsis immediately after emergency department triage. Scand J Trauma Resusc Emerg Med.

[48] Barnaby DP, Ferrick K, Kaplan DT (2018). Use of the low-frequency/high-frequency ratio of heart rate variability to predict short-term deterioration in emergency department patients with sepsis. Emerg Med J.

[49] Walker RG, Johnson MA, Culley LL (2018). Evaluation of physiologic alterations during prehospital paramedic-performed rapid sequence intubation. Prehosp Emerg Care.

[50] Davis WT, Thomas SH, Frakes MA (2022). Hemodynamic events during en route critical care for patients with traumatic brain injury. J Trauma Acute Care Surg.

[51] Reed MJ, Grubb NR, Lang CC (2019). Multi-centre randomized controlled trial of a smartphone-based event recorder alongside standard care versus standard care for patients presenting to the emergency department with palpitations and pre-syncope—the IPED (Investigation of Palpitations in the ED) study. EClinicalMedicine.

[52] Hansen LH, Ettrup-Christensen A, Bulow K (2019). Feasibility of continuous noninvasive arterial pressure monitoring in a prehospital setting: measurements during emergency transfer. Eur J Emerg Med.

[53] Koceska N, Koceski S, Kitanovski V (2020). Mobile wireless monitoring system for prehospital emergency care. Eur J Trauma Emerg Surg.

[54] Poncette AS, Spies CD, Mosch L (2022). A remote patient-monitoring system for intensive care medicine: mixed methods human-centered design and usability evaluation. JMIR Hum Factors.

[55] Sorkin A, Tsur AM, Nadler R (2022). BladeShield 101: a novel prehospital digital wearable combat casualty card. Isr Med Assoc J.

[56] Kuster M, Exadaktylos AK, Haberkern M (2019). Non-invasive cardiac output monitoring device “ICON” in trauma patients: a feasibility study. Eur J Trauma Emerg Surg.

[57] Yagi T, Nakamura M, Ishikawa T (2020). Usefulness of a new device to monitor cerebral blood oxygenation using NIRS during cardiopulmonary resuscitation in patients with cardiac arrest: a pilot study. Adv Exp Med Biol.

[58] Zanatta M, Borsato L, Manara R (2020). Ultrasound-guided chest compressions in out-of-hospital cardiac arrests. J Emerg Med.

[59] Kim D, Jin BT (2022). Development and comparative performance of physiologic monitoring strategies in the emergency department. JAMA Netw Open.

[60] Phan DT, Lee KH, Park HJ (2022). A flexible, wearable, and wireless biosensor patch with Internet of Medical Things applications. Biosensors (Basel).

[61] Walinjkar A (2018). A composite and wearable sensor kit for location-aware healthcare monitoring and real-time trauma scoring for survival prediction. Appl Syst Innov.

[62] Naregalkar A, Krishna GV (2019). Bio-signal system design for real-time ambulatory patient monitoring and abnormalities detection system.

[63] Zainuddin AA, Ismail W, Sulaiman N Patient monitoring system using computer vision for emotional recognition and vital signs detection.

[64] Nagayo AM, Garcia CC, Dadios EP An unmanned aerial robot and physiological data monitoring system integrated into a patient transport vehicle for emergency medical services and telehealth.

[65] Billis A, Koutras C, Sioulas N (2019). Towards the definition of an intelligent triage and continuous monitoring system for hospital emergency departments and clinics.

[66] Leenen JPL, Leerentveld C, van Dijk JD (2020). Current evidence for continuous vital signs monitoring by wearable wireless devices in hospitalized adults: systematic review. J Med Internet Res.

[67] Rebelo RAIB, Correlo VM, Reis RL (2021). An outlook on implantable biosensors for personalized medicine. Engineering.

[68] Totuk A, Bayramoglu B, Tayfur I (2023). Reliability of smartphone measurements of peripheral oxygen saturation and heart rate in hypotensive patients: measurement of vital signs with smartphones. Heliyon.

[69] Childs C, Barrow YM, Byers WF (2012). A systematic review on the role of extremity skin temperature as a non-invasive marker for hypoperfusion in critically ill adults in the intensive care setting. JBI Evid Synth.

[70] Kellett J, Rasool S, Green GC (2011). Comparison of the heart and breathing rate of acutely ill medical patients recorded by nursing staff with those measured over 5 minutes by a piezoelectric belt and ECG monitor at the time of admission to hospital. Resuscitation.

[71] Kumar SS, Dashtipour K, Abbasi QH (2021). A review on wearable and contactless sensing for COVID-19 with policy challenges. Front Commun Netw.

[72] Eastridge BJ, Holcomb JB, Shackelford S (2019). Outcomes of traumatic hemorrhagic shock and the epidemiology of preventable death from injury. Transfusion.

[73] Mohammed BG, Hasan DS (2023). Smart healthcare monitoring system using IoT. Int J Interact Mob Technol.

[74] Ramalingam GP, Pandian D, Batcha CFS (2024). IntelliCare: integrating IoT and machine learning for remote patient monitoring in healthcare: a comprehensive framework. J Cogn Hum Comput Interact.

[75] Kalasin S, Surareungchai W (2023). Challenges of emerging wearable sensors for remote monitoring toward telemedicine healthcare. Anal Chem.

[76] Boikanyo K, Zungeru AM, Sigweni B (2023). Remote patient monitoring systems: applications, architecture, and challenges. Sci Afr.

[77] Tseng L, Wong L, Otoum S (2020). Blockchain for managing heterogeneous internet of things: a perspective architecture. IEEE Netw.

[78] Kokez HADF On terrestrial and satellite communications for telecommunication future.

[79] Aghdam ZN, Rahmani AM, Hosseinzadeh M (2021). The role of the Internet of Things in healthcare: future trends and challenges. Comput Methods Programs Biomed.

